# Aneuploidy as a mechanism of adaptation to telomerase insufficiency

**DOI:** 10.1007/s00294-015-0559-x

**Published:** 2016-01-12

**Authors:** Caroline Millet, Svetlana Makovets

**Affiliations:** School of Biological Sciences, Institute of Cell Biology, University of Edinburgh, Roger Land Building Room 1.07, Alexander Crum Brown Road, Edinburgh, EH9 3FF UK

**Keywords:** Telomeres, Telomerase, Yeast, Aneuploidy, Adaptation, Telomerase insufficiency

## Abstract

Cells’ survival is determined by their ability to adapt to constantly changing environment. Adaptation responses involve global changes in transcription, translation, and posttranslational modifications of proteins. In recent years, karyotype changes in adapting populations of single cell organisms have been reported in a number of studies. More recently, we have described aneuploidy as an adaptation mechanism used by populations of budding yeast *Saccharomyces cerevisiae* to survive telomerase insufficiency induced by elevated growth temperature. Genetic evidence suggests that telomerase insufficiency is caused by decreased levels of the telomerase catalytic subunit Est2. Here, we present experiments arguing that the underlying cause of this phenomenon may be within the telomerase RNA TLC1: changes in the expression of TLC1 as well as mutations in the TLC1 template region affect telomere length equilibrium and the temperature threshold for the induction of telomerase insufficiency. We discuss what lies at the root of telomerase insufficiency, how cell populations overcome it through aneuploidy and whether reversible aneuploidy could be an adaptation mechanism for a variety of environmental stresses.

## Introduction

Eukaryotic chromosomes are linear DNA molecules which due to the end replication problem cannot be copied fully at both ends by the conventional replication mechanism. To compensate for the DNA loss at chromosome ends most eukaryotes evolved telomerase, a reverse transcriptase enzyme which consists of RNA and protein components and uses the RNA as a template to extend DNA 3′ ends (Greider and Blackburn 1985, 1987). These chromosome ends, telomeres, consist of noncoding repetitive DNA sequences rich in G-C content, for example (TG_1–3_TG)_*n*_ in *Saccharomyces cerevisiae* (Shampay et al. 1984) and (TTAGGG)_*n*_ in mammals (Moyzis et al. 1988). All the telomeres within a cell are homologous to one another and some telomeres also share homology at sub-telomeric regions. Telomerase loss often causes delayed rather than immediate lethality (Lundblad and Szostak 1989), a unique phenotype explained by the fact that cells normally maintain telomeres longer than the minimum length necessary for their viability. After telomerase loss, it takes a succession of replication cycles for telomeres to become critically short, cause cell cycle arrest and, eventually, cell death (AS and Greider 2003; Le et al. 1999; Lundblad and Blackburn 1993; Lundblad and Szostak 1989; Teng and Zakian 1999).

Telomerase loss has been well-studied in budding yeast *S. cerevisiae*. Loss of either telomerase RNA TLC1 or one of the essential protein components of the telomerase complex Est1, Est2, or Est3 results in gradual telomere shortening over 40–60 generations, followed by cell viability crisis and generation of rare survivors which maintain their telomeres via recombination between either telomeric or sub-telomeric repeats (AS and Greider 2003; Le et al. 1999; Lundblad and Blackburn 1993; Lundblad and Szostak 1989; Singer and Gottschling 1994; Teng and Zakian 1999). We have recently reported a phenomenon of telomerase insufficiency in *S. cerevisiae*: when grown at higher temperatures, yeast encounter a telomere maintenance problem due to limited levels of the telomerase catalytic subunit Est2 (Millet et al. 2015). Interestingly, this telomerase-positive yeast with insufficient amounts of telomerase behaves differently from telomerase-negative cells: instead of switching to recombination-dependent telomere maintenance they can generate survivors monosomic for chromosome VIII. The chromosome VIII monosomy leads to upregulation of the telomerase components and suppression of telomerase insufficiency (Millet et al. 2015). Below, we present additional experimental data as an extension of our initial investigation and discuss different aspects of our findings and their relevance to other studies on telomere biology, genome stability, and mechanisms of adaptation to changing environment.

## Materials and methods

### Strains and media

All the strains used in the study are of *S. cerevisiae* A364 background. Strain passaging for telomere length analysis was done on plates with standard rich media (YPD: 2 % peptone, 1 % yeast extract, 2 % glucose, and 2 % agar) with additional 0.01 % adenine, 0.01 % tryptophan, and 0.003 % uracil.

### Plasmid construction

pYT84 is based on the integration vector pRS306 and contains the *BamH*I-*Xho*I fragment with *TLC1* with the endogenous promoter and transcriptional terminator as described before (Prescott and Blackburn 1997). To construct a plasmid with *TLC1* under the *CLN2* promoter (pYT294), Acc65I-BamHI fragment of pYT84 was replaced with an *Acc*65I-*BamH*I digested PCR product containing *CLN2* promoter. The following primers were used in the PCR reaction: CCCC**GGATCC**CCGAAAACGGAAATCATCGC and CCCC**GGTACC**TATCTTCCTCTCTAGTTTTATTTGTCTGTCGTTAAATTTAATGAATG (restriction sites are in bold). To make a derivative of pYT294 with the *CLN2* promoter truncated (pYT297), pYT294 was digested with *BamH*I and *Sph*I and the ends were ligated using a *BamH*I-*Sph*I linker.

### Southern blotting

Total genomic DNA was digested overnight with either *Xho*I or *Kpn*I and the samples were resolved on 0.85 % agarose gels and transferred to a charged Nylon membrane using capillary transfer. A P-32 end-labelled telomere-specific oligo (CACCCA)_4_ was used to visualise all the telomeres in *Xho*I digested samples, while a Y′-specific KL1 probe (Makovets et al. 2004) was used to detect Y′ telomeres in the experiments involving *Kpn*I digests.

### Array comparative genome hybridisation (aCGH)

Total genomic DNA was purified from yeast cells as described before (Makovets et al. 2008). A diploid control strain was used as a reference. DNA samples were submitted to Oxford Gene Technology (OGT) for labelling and hybridisation to custom designed 135K arrays tiling 16 chromosomes (non-repetitive sequences), mitochondrion and Y′ repeats of *S. cerevisiae*.

## Results and discussion

### What causes telomere maintenance problems at higher growth temperatures?

**Fig. 1:**
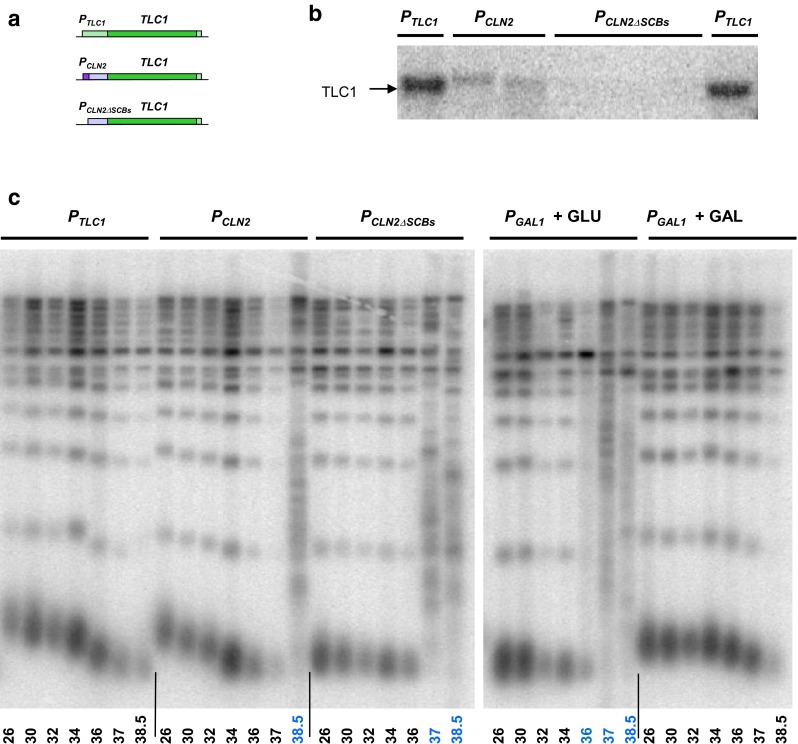
The effect of *TLC1* expression on temperature-induced telomerase insufficiency. **a** A schematic of three constructs used for *TLC1* expression: from its own promoter shown in *light green* (*top*), from 623-bp long *CLN2* promoter (*light purple*) with the transcriptional activator binding sites (SCBs, depicted in *dark purple*) present (*middle*), and a 506 bp *CLN2* promoter derivative without SCBs (*bottom*). **b** Relative levels of TLC1 RNA assayed by Northern blotting. Equal amount of total RNA was loaded per each* lane*. Multiple independently constructed clones are shown for TLC1 expressed from two different versions of *CLN2* promoter (promoters used labelled *above* the corresponding* lanes*). **c** Telomere length analysis by Southern blotting in cells grown for ~100 generations (5 passages) at the temperatures indicated below the corresponding* lanes*. Total genomic DNA was digested with *Xho*I and the blots were probed with a telomere-specific probe. Samples with the temperatures in blue show telomere patterns characteristic of recombination-dependent telomere maintenance. Promoters used for *TLC1* expression in these cells are stated above the corresponding gel images

What is the key cause for temperature-triggered telomerase insufficiency in yeast? We have identified Est2 as a telomerase component which is present at a vastly decreased level at higher growth temperatures, but as little as a second copy of *EST2* expressed from its own promoter is sufficient for suppression of telomerase insufficiency to maintain short telomeres. Why is Est2 affected by growth temperature? It has been shown that the Est2 levels in vivo are dependent on the presence of TLC1 (Tucey and Lundblad 2014): perhaps Est2 is unstable unless it is bound to TLC1. Therefore, understanding how high temperature affects TLC1 may hold the key to elucidating the variations in the Est2 levels as a result of dynamic changes in telomerase as a complex and uncovering the true causes of telomerase insufficiency. While the problem of protein folding (or rather unfolding) in cells exposed to higher temperatures is well-documented, little is known about how elevated temperature affects base pairing in RNA in eukaryotes in vivo. Folding of RNA molecules with short stretches of dsRNA is likely to be destabilised at higher temperatures. In fact, prokaryotes use dsRNA melting in response to heat, so-called RNA thermometers, to coordinate gene expression with growth temperature (Krajewski and Narberhaus 2014). The RNA component of yeast telomerase TLC1 contains multiple short stem-loops and a pseudoknot required for its function (Niederer and Zappulla 2015). Higher growth temperature might destabilise the dsRNA in the TLC1 regions required for Est2 binding (Chappell and Lundblad 2004) and therefore less Est2 would be in complex with TLC1 at higher temperatures and more Est2 might be degraded due to its unbound state. Unfortunately, experiments involving manipulations of *TLC1* nucleotide sequences to stabilise the RNA would not be straight forward as such sequence changes are likely to affect the recognition of TLC1 by Est2. However, it is possible to manipulate the expression of *TLC1* to ask if the levels of telomerase RNA alone would affect the temperature-induced telomerase insufficiency, perhaps by affecting the amount of TLC1–Est2 complex. Turning down the levels of TLC1 by expressing the gene from the well-studied *CLN2* promoter either with or without the Swi4/Swi6-dependent transcriptional activation (Bai et al. 2010) led to replicative senescence even at lower growth temperatures (Fig. 1a–c). While the lower levels of TLC1 expressed from the two versions of the *CLN2* promoter were sufficient to maintain shorter, stable telomeres at lower temperatures a transition to telomerase-independent telomere maintenance (Type II) was required for *P*_*CLN2*_-*TLC1* cells to survive at higher temperatures (Fig. 1c, left panel). In contrast, overexpression of *TLC1* from an induced *GAL1* promoter stabilised the telomere length at up to 34 °C (Fig. 1c, right panel). Therefore, manipulating the levels of TLC1 alone affects not only the telomere length equilibrium, but also the temperature at which yeast experience telomerase insufficiency and replicative senescence. However, in most cases the survivors maintained their telomeres via recombination, suggesting that telomerase insufficiency in cells with downregulated TLC1 could no longer be suppressed by aneuploidy at 38.5 °C. It would be interesting to analyse cells with the turned down TLC1 expression at temperatures close to the ones triggering senescence in order to test if chromosome VIII monosomy could allow viability crises to be bypassed by suppressing telomerase insufficiency.

**Fig. 2:**
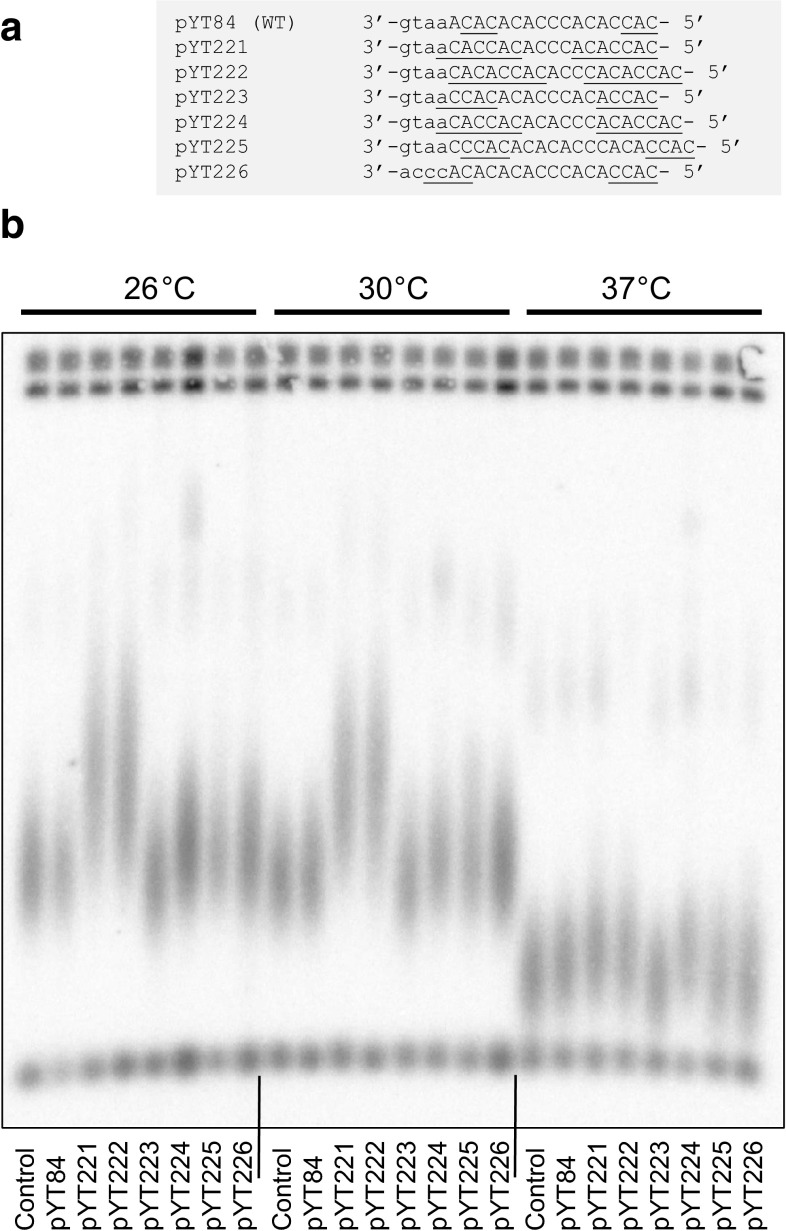
Mutations in the TLC1 template region affect telomere length equilibrium. **a** Template variants used in the study. Homologies between the 3′ and 5′ regions within each template are u*nderline*d. All the constructs were within a complete *TLC1* gene cloned under its own promoter into an integration vector pRS306 (the plasmid names are shown on the *left*) and integrated into *tlc1Δ* cells at the endogenous *TLC1* locus. **b** Telomere length in different *tlc1* template mutants grown for ~100 generations at the temperatures indicated above the image. Total genomic DNA samples were digested with *Kpn*I and the blots were hybridised to a Y′-specific probe. The “Control” strain in the left-most* lane* of each sample set corresponds to yeast with the intact *TLC1* locus. The rest of the strains, each contains a pYT plasmid (indicated *below* the* lane*) with a wild-type (pYT84) or mutated (pYT221–226) *TLC1* integrated at the *TLC1* locus where the endogenous copy of *TLC1* has been deleted prior to the plasmid integration

The annealing of the 3′ end of a telomere to the template region of TLC1 prior to telomere extension by telomerase is limited to a few nucleotides and therefore might also be affected by temperature. Due to redundancy of telomeric repeats in *S. cerevisiae* it is hard to predict how many nucleotides could be involved in the telomere-TLC1 pairing. We constructed a series of TLC1 template derivatives with the general strategy to (a) increase the sequence similarity between the 3′- and the 5′-ends of the template so that if the template is copied exactly then the annealing prior to the next round of telomere extension would be optimal; (b) maximise the C-content in the template region (to promote telomere–template pairing) without affecting the telomeric repeat consensus (Fig. 2a). Five out of the six mutated template variants resulted in longer telomeres at 26, 30, and 37 °C (Fig. 2b) suggesting that the efficiency of telomere-TLC1 annealing might be an important factor playing a role in telomere length equilibrium. Alternatively, the modified TLC1 template regions might have resulted in changes in telomeric DNA sequence leading to altered spacing between Rap1 binding sites, and therefore re-equilibration of telomeres at an increased length. Further experiments involving telomere sequencing would be needed to distinguish between the possible explanations.

### How does chromosome VIII aneuploidy suppress telomerase insufficiency?

**Fig. 3:**
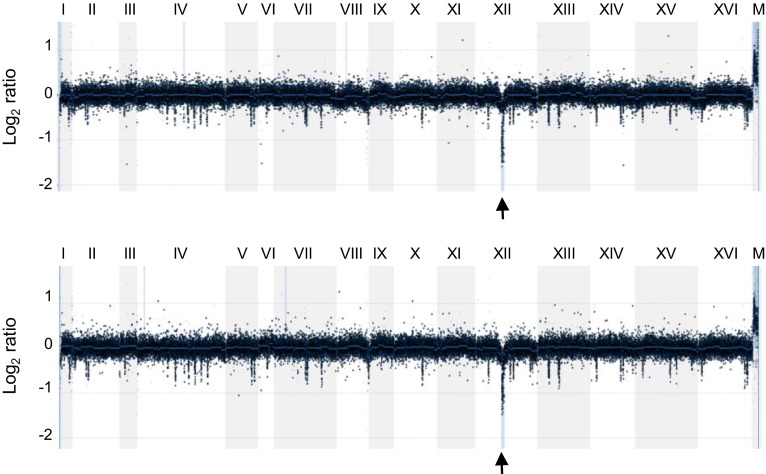
aCGH analysis of two haploid telomerase insufficiency survivors with decreased rDNA. The relative amount of DNA in the survivors was normalised against an isogenic wild-type haploid stain. Notice that both survivors show more than a twofold decrease in the copy number of rDNA repeats (indicated by *black arrow* below each aCGH profile) which are located on chromosome XII. Chromosome number and mitochondrial DNA (M) are indicated above each profile

In order to understand how yeast can survive telomerase insufficiency, we have analysed a number of survivors adapted to continuous growth at 38.5 °C. The survivors can be divided into two major categories: (a) haploid-like survivors which show ~1n DNA cont5ent by FACS analysis and in most cases euploidy in aCGH (array Comparative Genome Hybridisation) analysis and (b) diploid-like survivors which are ~2n by FACS but monosomic for chromosome VIII as revealed by aCGH. A few chromosome VIII monosomics also had a single copy of chromosome I, but the copy number of this chromosome did not correlate with the suppression of telomerase insufficiency. Therefore, one of the mechanisms that *S. cerevisiae* use to suppress telomerase insufficiency is a karyotype change: populations of haploid yeast experiencing viability crises due to critically short telomeres generate survivors monosomic for chromosome VIII. Extensive deletion analysis of chromosome VIII resulted in identification of four genes important for overcoming telomerase insufficiency: *PRP8*, *UTP9*, *KOG1*, and *SCH9*. All the proteins encoded by these genes are connected to ribosome production. Prp8 is a splicing factor (Vijayraghavan et al. 1989): in budding yeast most genes do not contain introns, but the majority of ribosomal protein (RP) genes require splicing. Utp9 is indispensable for the processing of rRNA (Dragon et al. 2002), while Kog1 and Sch9 function in the TOR-pathway which has been previously implicated in the telomere length homeostasis in yeast (Ungar et al. 2011) through its effect on Ku complex required for nuclear localisation of telomerase (Pfingsten et al. 2012). However, the major role of TOR is in regulation of genes required for ribosome production in response to nutrition availability (Broach 2012). Not surprisingly, chromosome VIII aneuploids have a decreased abundance of both ribosomal proteins and mature rRNA, accompanied by increased abundance of the telomerase components TLC1, Est1 and Est3. The haploid-like survivors generated in our study have not been analysed in detail, but some of them demonstrated a decreased copy number of ribosomal DNA repeats (Fig. 3). Therefore, downregulation of ribosome production might be relevant to the suppression of telomerase insufficiency in the haploid survivors too.

We proposed a mechanism for upregulation of telomerase in cells monosomic for chromosome VIII. Ribosome production is responsible for the vast majority of the workload for yeast transcription, splicing, and translation machineries (for a comprehensive review see Warner 1999). Therefore, downregulation of ribosome production at the transcriptional level alone should free up significant translation capacities which in turn could be used to synthesise more non-ribosomal proteins, including the telomerase subunits Est1 and Est3. Interestingly, the steady-state levels of Est2 are not increased in the chromosome VIII aneuploids (Millet et al. 2015) suggesting that there might be a mechanism co-ordinating the amount of Est2 with cell growth. The increased levels of Est1, Est3, and TLC1 in the aneuploids were necessary for the suppression of telomerase insufficiency, presumably by contributing to increased levels of the complete telomerase complex and/or improved recruitment of telomerase to telomeres via the Est1–Cdc13 pathway. Downregulation of ribosome biogenesis in the aneuploids also results in a noticeably slower growth and longer cell cycle which might result in a longer time window for telomerase to operate on telomeres during the late S-phase. Further experiments are under way to gain molecular insights into the mechanisms of suppression of telomerase insufficiency in cells monosomic for chromosome VIII.

### Is chromosome VIII monosomy similar to ESR?

While the upregulation of telomerase components allows cells to overcome telomere-related stress and suppress telomerase insufficiency, the downregulation of ribosome production as a cellular reaction is not unique to telomere stress but a signature of environmental stress response (ERS), a common response to diverse stresses such as oxidative damage, heat shock, starvation, DNA damage, high osmolality, etc. (reviewed in Gasch 2007). Mammalian telomerase expressed in yeast has also been suggested to protect their genome from oxidative stress (Simonicova et al. 2015). How much do chromosome VIII aneuploids resemble cells with ESR activated? A direct, side-by-side comparison will be required to answer this intriguing question, as extended similarity between the two conditions would suggest that chromosome VIII monosomy might be a means to keep ESR activated constitutively through a karyotype change, avoiding the persistent sensing and signalling required to maintain ESR active. The energy costs might be lower in the case of aneuploidy, as maintaining active ERS signalling required for the transcriptional response in euploids under stress would not be necessary in the chromosome VIII aneuploids.

Rap1 may represent a key link between cell growth, environmental stress response and telomeres. Rap1 is the major telomere-binding protein important for telomere length equilibrium (Shore 1997), but it also binds numerous promoter regions throughout the genome in order to regulate transcription of RP genes, translation factors and highly transcribed glycolytic genes (Warner 1999). Rap1 is also required for telomere response to caffeine and ethanol: its levels are downregulated in response to stress and this change results in longer telomeres (Romano et al. 2013). Although the levels of Rap1 were similar in diploids and chromosome VIII aneuploids (Millet and Makovets, unpublished results), it is unknown if Rap1 binding at the promoters and telomeres is affected by chromosome VIII monosomy.

### Reversible aneuploidy as an adaptation mechanism

It has been shown that increased ploidy improves adaptation abilities of cell populations and that specific aneuploidies confer growth advantages in particular conditions (Reviewed in Chen et al. 2012). However, aneuploids normally have a growth disadvantage outside of these conditions. Therefore, if aneuploidy were used by some species as an adaptation mechanism in response to changing environment it would be important that when the stress is removed the populations could switch back to euploidy in order to maintain robust growth and proliferation. This is exactly the type of behaviour previously reported for chromosome III disomics (Yona et al. 2012), and now we have documented it for chromosome VIII monosomics (Millet et al. 2015). The chromosome VIII aneuploidy suppresses telomerase insufficiency at 38.5 °C, but it also leads to slower growth at 30 °C. However, at 30 °C the aneuploid populations generate colonies of fast growing euploids which take over the population in a short period of time. Perhaps, gaining a second copy of chromosome VIII is a stochastic process involving chromosome mis-segregation which might also be affected by aneuploidy, i.e., the aneuploids might have a higher rate of mitosis errors allowing the population to generate euploids with a relatively high frequency to simplify the transition back to euploidy. One can envision a yeast culture as a bi-modal (or even tri-, or tetra-modal and so on) population where cells with different karyotypes might be generated all the time, assuming the rate of chromosome mis-segregation is high enough. Such a population might have increased capabilities to adapt to a whole spectrum of environmental stresses through the differential survival of cells with variable karyotypes.

### What limits cell growth at higher temperature and how do cells adapt?

**Fig. 4:**
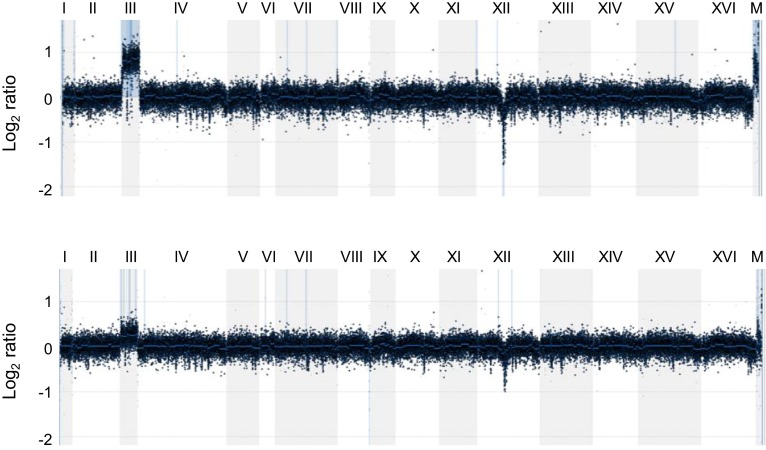
aCGH analysis of two haploid telomerase insufficiency survivors with increased signal for chromosome III. The relative amount of DNA in the survivors was normalised against an isogenic wild-type haploid stain. Notice that the profile on the top has a twofold increase in chromosome III signal indicating chromosome III disomy, while the chromosome III increase in the other profile is less than twofold. This may indicate a mixture of cells in the population: some are haploid and others are disomic for chromosome III. Both survivors also show a decrease in the copy number of rDNA repeats on chromosome XII. Chromosome number and mitochondrial DNA (M) are indicated above each profile

In a lab evolution study, the Pilpel group identified chromosome III trisomy as a result of adaptation of diploid *S. cerevisiae* S288c (a commonly used laboratory strain) to growth at 39 °C (Yona et al. 2012). In our hands, yeast of the S288c strain background can tolerate higher growth temperatures than those of A364 used in our studies. S288c also have slightly longer telomeres and therefore it is possible that telomere maintenance was not a factor in the lab evolution study. However, among a limited number of haploid-like survivors analysed by aCGH, we also found 2 clones (out of 13 analysed) with chromosome III disomy (Fig. 4) which may provide a similar adaptation mechanism to that described for the chromosome III trisomy in diploids, namely, increased expression of some genes on chromosome III due to the added gene dosage.

In another lab evolution study designed to isolate an industrial *S. cerevisiae* strain with robust growth at 40 °C, Nielsen’s lab-isolated mutants with altered sterol metabolism and showed that changes in sterol composition in yeast cellular membranes might confer thermotolerance (Caspeta et al. 2014). Although these experiments have been done in settings completely different from ours (growth media and conditions, strain background, etc.) it would be interesting to find out how the lab-evolved strains maintain their telomeres: is there enough telomerase for this particular strain to grow in these particular conditions or are they telomerase-independent survivors maintaining their telomeres via recombination?

### Does adaptation spectrum define the range of telomere length homeostasis of a species?

Telomere length has been established for a variety of organisms and it ranges from a few hundred base pairs to hundreds of kilobase pairs in some plants. Why do different species have different telomere lengths? It is believed that in larger sized animals which downregulate telomerase expression post-embryonically, the telomere length set at birth should be sufficient to provide enough replication potential for normal growth and development, yet become a proliferation-restricting factor for cancer progression (Gomes et al. 2010). In simple unicellular organisms such as yeast telomeres are rather short and require constitutive expression of telomerase for their maintenance. Telomere length is known to be affected by a variety of growth factors present in the media (Romano et al. 2013). Both in budding yeast *S. cerevisiae* and fission yeast *Schizosaccharomyces pombe* telomere length is also temperature dependent: cells grown at higher temperatures have shorter telomeres (Millet et al. 2015). While *S. pombe* cultured close to their maximum growth temperature initiate recombination at sub-telomeric regions, *S. cerevisiae* show a viability crisis caused by telomerase insufficiency. Is it a coincidence that telomere maintenance becomes an issue only at temperatures very close to the maximum temperature suitable for the growth of an organism? We propose that the telomere maintenance machinery may have co-evolved along with the species’ ability to adapt to a variety of conditions, so that while telomeres may undergo length changes in response to different stress factors, they would remain functional under the range of conditions to which cells are able to adapt to maintain proliferation of cell populations. In those rare situations when environmental stress drives telomeres out of telomere length homeostasis, there is a backup mechanism allowing the population to survive through aneuploidy.
